# Enhanced Mechanical and Thermal Properties of Chain-Extended Waterborne Polyurethane Coatings with Cellulose Acetate Butyrate

**DOI:** 10.3390/polym14194062

**Published:** 2022-09-27

**Authors:** Yong-Rok Kwon, Hae-Chan Kim, Jung-Soo Kim, Ju-Hee So, Young-Wook Chang, Dong-Hyun Kim

**Affiliations:** 1 Material & Component Convergence R&D Department, Korea Institute of Industrial Technology (KITECH), Ansansi 15588, Korea; 2 Department of Material Chemical Engineering, Hanyang University, Ansansi 15588, Korea

**Keywords:** waterborne polyurethane, cellulose acetate butyrate, chain extender, tensile strength, microphase separation

## Abstract

A series of waterborne polyurethane (WPU) dispersions were prepared by chain-extending a prepolymer made of polyester diol, isophorone diisocyanate, and dimethylol propionic acid using cellulose acetate butyrate (CAB). The particle size and viscosity of the WPU dispersion were measured. In addition, we investigated the effects of CAB on the thermal, mechanical, and optical properties of WPU films. The use of CAB effectively improved the crosslinking degree of the WPUs, increasing the thermal stability and water resistance of the corresponding films. In particular, CAB increased the tensile strength of the WPU films up to 67%, while maintaining their elongation at break unchanged. In addition, CAB improved the optical transmittance by reducing the microphase separation between the soft and hard segments of PU. The rough surface structure of the WPU films formed by CAB led to improved matting properties.

## 1. Introduction

Growing concerns about human health risks and environmental pollution have limited the application of most organic solvent-based coatings that release large amounts of volatile organic compounds (VOCs) [1]. Therefore, waterborne formulations of polyacrylates, polyurethanes, epoxy resins and alkyd resins with advantages such as low toxicity, flammability and VOC emission are of great interest in coating applications [2]. However, conventional waterborne coatings in which polymers are emulsified using surfactants have poor properties and cause environmental pollution [3]. As a result, the development of self-emulsifying waterborne coatings including a hydrophilic segment in a polymer backbone has received wide attention in both scientific and practical contexts [4].

Waterborne polyurethanes (WPUs) have become important industrial coating materials due to excellent properties such as flexibility, impact resistance, transparency, abrasion resistance, and low VOC content [5]. Synthetic routes for WPUs have been reported in various studies; most WPUs are prepared by emulsifying a prepolymer synthesized with polyol, isocyanate, and an internal emulsifier in water, followed by a chain extension step [6].

In particular, chain extension is essential to improve the mechanical strength of WPU films and expand their application scope. However, because most chain extenders are based on petrochemicals, several studies have been conducted to replace them with renewable resources. Wu et al. increased the stiffness and strain of nanocomposites by incorporating microcrystalline cellulose (serving as a chain extender) into WPU [7]. Travinskaya et al. improved the tensile strength and biodegradability of polyurethane films by introducing aqueous starch solutions into the polyurethane dispersion and chain extension steps [8]. Lee et al. prepared a WPU film with improved self-healing efficiency using chitosan as a chain extender [9]. However, the introduction of a high-molecular weight chain extender such as a natural polymer after the water dispersion process of the prepolymer may cause agglomeration or a non-uniform reaction on the surface of the agglomerated or dispersed particles [10].

Cellulose acetate butyrate (CAB) is a cellulose derivative containing acetyl and butyryl groups that can be used in the coating industry. CAB has the advantages of good surface gloss and high transparency, as well as good solubility, compatibility, hardness, moisture resistance, and cold resistance [11]. The unsubstituted free hydroxyl group of CAB can covalently bond with the NCO group of the prepolymers, suggesting that CAB can act as a chain extender in polyurethane synthesis. In addition, the multi-functionality of CAB can provide a crosslinking point to compensate for the deteriorated water resistance of WPU film caused by hydrophilic functional groups introduced for water dispersion of PU. CAB can be easily incorporated into the WPU system by a uniform reaction, owing to its high mixability with the polyurethane prepolymer. However, although the effect of chain extension using CAB on the WPU preparation is a very interesting subject, only limited information is available on this issue.

In this work, CAB, a renewable resource, was used to obtain CAB-modified WPUs with improved thermomechanical properties and water resistance. For this purpose, an NCO-terminated prepolymer was synthesized using polyester diol, isophorone diisocyanate (IPDI), and 2,2-dimethylolpropionic acid (DMPA); the prepolymer was chain-extended with CAB, followed by dispersion in water to prepare the WPUs. Fourier-transform infrared (FTIR) spectroscopy was used to characterize the structure of the CAB-integrated PU materials. In addition, we investigated the physicochemical, thermal, and mechanical properties of a series of WPU films with different CAB contents to evaluate the applicability of CAB as a chain extender.

## 2. Materials and Methods

### 2.1. Materials

Poly(1,4-butylene adipate) (PBAT, *M*_n_ = 1000 g/mol, BASF, Ludwigshafen, Germany), IPDI (Sigma-Aldrich, St. Louis, MI, USA), and DMPA (Sigma-Aldrich, St. Louis, MI, USA) were chosen as polyol, isocyanate, and internal emulsifier, respectively, for the synthesis of an NCO-terminated prepolymer. PBAT, IPDI, and DMPA were dried under vacuum at 60 °C for 12 h prior to their use. Dibutyltin dilaurate (DBTDL, Sigma-Aldrich, St. Louis, MI, USA) was used as a catalyst, whereas trimethylamine (TEA, Sigma-Aldrich, St. Louis, MI, USA) served as a neutralizer. CAB (*M*_n_ = 12,000 g/mol, 17.5% acetyl, 32.5% butyryl, and 1.3% hydroxyl, Sigma-Aldrich, St. Louis, MI, USA) and ethylene diamine (EDA, Sigma-Aldrich, St. Louis, MI, USA) were used as chain extenders. The 1-methyl-2-pyrrolidone (NMP) was purchased from Samchun Chemical in South Korea.

### 2.2. Preparation of WPU Dispersions and Films

**Figure 1:**
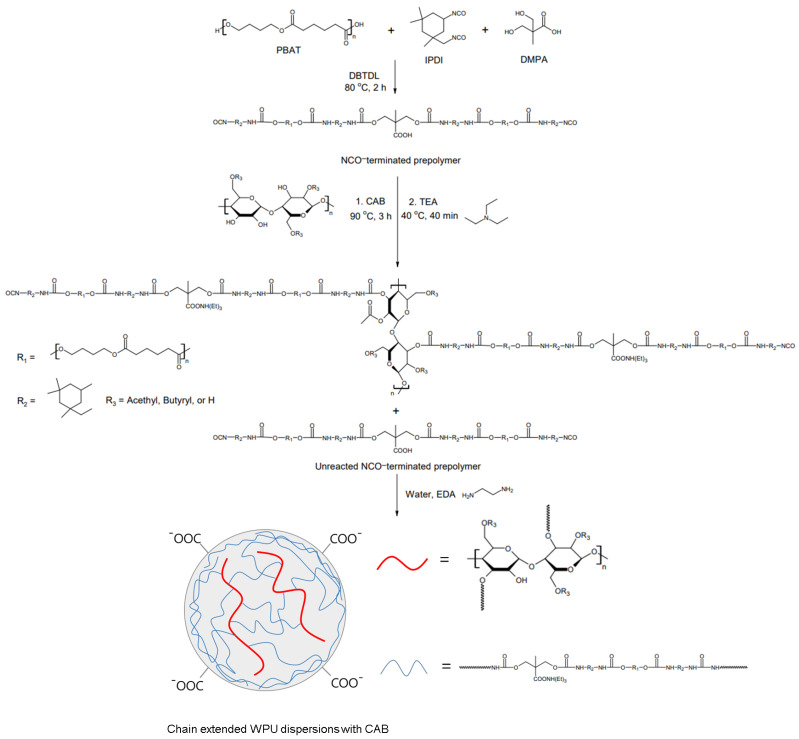
Preparation of chain-extended WPU dispersions with CAB.

The schematic procedure for the preparation of WPU dispersions with different CAB contents is shown in Figure 1. The reaction was carried out in a 500 mL four-necked flask equipped with a mechanical stirrer, thermometer, condenser, and nitrogen inlet. First, PBAT, IPDI, and DMPA dissolved in NMP were placed into the reactor and reacted at 90 °C for 1 h. Then, DBTDL was added and further reacted for 3 h. After that, CAB dissolved in acetone was added for the chain extension step, and the reaction was continued under stirring until the theoretical NCO content was reached. After the reactant was cooled to room temperature, the carboxyl groups were neutralized with TEA in an equimolar amount to DMPA for 40 min. After that, the stirring speed was increased, and calculated amounts of distilled water and EDA were added dropwise to the reactor. EDA was used for additional chain extension and removal of free isocyanates. The mixture was dispersed and reacted at room temperature for 1 h to obtain a milky white WPU dispersion. All samples exhibited a solids content of 30.4 ± 1.2 wt.%.

Table 1 lists the formulations used for the preparation of the WPU dispersions. The WPU films were obtained after casting the prepared WPU dispersions, drying at room temperature for 24 h, and further drying at 80 °C for 24 h.

The theoretical value of NCO% was calculated using Equation (1):

(1) $$\mathrm{NCO}\%=\frac{\left(M_{NCO}-M_{OH}\right)\times 42}{W_{NCO}+W_{OH}}\times 100,$$

where *M_NCO_* and *M_OH_* are the mole number of the NCO and OH group, respectively. *W_NCO_* is the weight of IPDI. *W_OH_* is the weight of compositions with OH group. The experimental NCO% was determined by standard di-*n*-butylamine titration method.

### 2.3. Characterization

#### 2.3.1. Characterization of WPU Dispersions

The mean particle size and distribution of the WPU dispersions were analyzed by a particle size analyzer (LS 13 320, Beckman Coulter, Brea, CA, USA). The particle size dispersion (PDI) was calculated using Equation (2):

(2) $$\mathrm{PDI}=\frac{D_{90}-D_{10}}{D_{50}},$$

where *D*_10_, *D*_50_, and *D*_90_ represent the cumulative distribution of the diameters for 10%, 50%, and 90% of the particles, respectively [12].

The viscosity of the WPU dispersions was measured with a Brookfield viscometer (DV2TLV, AMETEK Brookfield, Berwyn, PA, USA). Approximately 15 mL of WPU dispersions were placed in a cylindrical sample chamber and measurements were performed using an LV-3 spindle at 25 °C and 100 rpm. The Brookfield viscosity was calculated as the average of three experimental measurements.

The solid content of the WPU dispersions was determined with a halogen moisture analyzer (MAC 50/WH, RADWAG, Random, Poland). The WPU dispersions (1 g) were placed in an aluminum pan and the solvent was evaporated at 105 °C until no change in weight was observed.

The storage stability of the WPU dispersions was determined based on whether coagulation and sedimentation occurred. The WPU dispersions were sealed and kept still under natural conditions, and their appearance was observed for 6 months.

#### 2.3.2. Characterization of WPU Films

The FTIR spectra of the WPU films were recorded with a FTIR spectrometer (Nicolet, Thermo Fisher Scientific, Waltham, MA, USA). All samples were scanned 64 times at a resolution of 8 cm^−1^ in the range of 400–4000 cm^−1^.

The thermal behaviors of the WPU films were investigated using differential scanning calorimetry (DSC, Q100, TA Instruments, New Castle, DE, USA). Two heating and cooling cycles were carried out in the temperature range between −90 and 100 °C, under N_2_ atmosphere at a heating rate of 10 °C/min. The glass transition temperature (*T*_g_) was determined at the mid-point of the glass transition after the second scan.

The thermal stability of the WPU films in temperature range of 30–600 °C was studied using thermogravimetric analysis (TGA, TA-2000, Dupont Co., Wilmington, NC, USA) at a heating rate of 10 °C/min under an N_2_ atmosphere.

The tensile measurement was performed using a Tinius Olsen 5ST. Tensile specimens size was 40 × 10 × 0.8 mm. The specimens were elongated at 10 mm/min.

The surface morphologies of the WPU films were examined using scanning electron microscopy (SEM, SU8010, Hitachi, Tokyo, Japan). Gold-coated WPU films were analyzed at an acceleration voltage of 5 kV.

The contact angles of the WPU films were acquired using a contact angle goniometer (JC2000C1, Shanghai Zhongchen Digital Technical Equipment Ltd., Shanghai, China). Then, 5–10 µL of distilled water was pumped onto WPU film. All results were reported as the mean of five measurements.

To measure the swelling ratio of the WPU films, the samples were swelled in water at room temperature for 24 h. After removing water from the sample surface using a paper towel every 2 h, the mass was measured. For all samples, the swelling ratio was calculated using Equation (3):

(3) $$\mathrm{Swelling}\;\mathrm{ratio}=\frac{\omega_{1}-\omega_{0}}{\omega_{0}}\times 100,$$

where *ω*_1_ and *ω*_0_ are the weights of the swollen and dried WPU film, respectively.

The optical properties of the WPU films were measured using an UV-visible spectrophotometer (Mega-800, Scinco, Seoul, South Korea) in the wavelength range of 400–800 nm. The gloss of the WPU films was assessed using a gloss meter (AG-4563, BYK-Gardner, Geretsried, Germany). All measurements were carried out with a 60° incidence angle.

## 3. Results and Discussion

### 3.1. Structure Analysis

**Figure 2:**
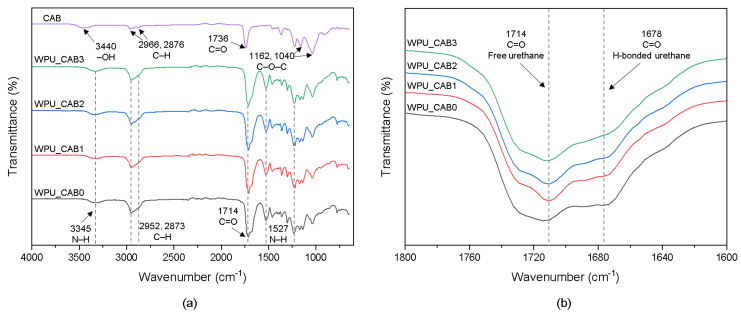
FTIR spectra (**a**) in the overall frequency range and (**b**) in the C=O stretching region of WPU films with different CAB contents.

The chemical structures of CAB and WPU films were confirmed by FTIR analysis (Figure 2). As shown in Figure 2a, CAB exhibited a broad and strong absorption peak at 3440 cm^−1^ due to the stretching vibration of -OH groups. The doublet peaks at 2966 and 2876 cm^−1^ are attributed to the antisymmetric and symmetric stretching vibrations of the methylene group [13]. The strong absorption peak at 1736 cm^−1^ can be assigned to the C=O stretching vibration [14]. The peaks at 1162 and 1040 cm^−1^ correspond to the stretching vibration of the unique C–O–C ether structure in CAB [15].

All WPU films showed a broad absorption peak near 3345 cm^−1^, corresponding to the N–H stretching vibration of the carbamate group. The peaks around 1714 and 1527 cm^−1^ are attributed to the C=O and N–H stretching vibrations, respectively [16]. C–H symmetric and asymmetric stretching vibrations of the CH_2_ group were observed at 2952 and 2673 cm^−1^, respectively. The NCO peak near 2265 cm^−1^ was not observed [17].

To analyze the C=O characteristic bands of the WPU films, enlarged views of the spectra are shown in Figure 2b. The band at ~1720 cm^−1^ is related to the free urethane C=O groups, whereas the shoulder around 1700 cm^−1^ can be attributed to hydrogen-bonded urethane C=O groups [18]. As the CAB content increased, the intensity of the shoulder corresponding to hydrogen-bonded C=O decreased. This means that the CAB incorporation hindered the hydrogen bonding between the urethane groups. In other words, the chain extension by CAB limited the mobility of the molecule, thereby increasing the amount of isolated hard segments trapped in the soft segment domain.

### 3.2. Properties of WPU Dispersions

Some properties of the WPU dispersions are shown in Table 2. The experimental solid contents for all WPU dispersions were similar to the theoretical values and ranged from 29 to 32 wt.%. In addition, all WPU dispersions remained stable without sediment for more than 6 months.

**Figure 3:**
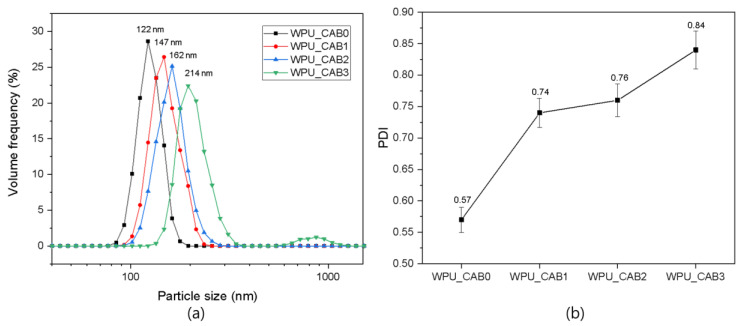
(**a**) Particle size distribution and (**b**) PDI of WPU dispersions with different CAB contents.

The average particle size and size distribution of WPU dispersions with different CAB contents were characterized using a laser diffraction particle size analyzer. Figure 3a shows the effect of the CAB content on the particle size of the WPU dispersions. As the CAB content increased, the particle size of the WPU dispersion also increased, likely due to the increase in the molecular weight and viscosity of the prepolymer upon chain extension. In the aqueous dispersion step, the larger viscosity of the dispersed (prepolymer) phase compared to the continuous (aqueous) phase led to a smaller deformation of the dispersed phase, resulting in a larger particle size and a wider distribution [19]. In the case of WPU_CAB3, the intermolecular entanglement increased due to excessive chain extension, resulting in non-uniform particles with a size of about 850 nm. The PDI of all samples was found to be within the range of 0.57 to 0.84; a PDI lower than 1 indicates a narrow particle size distribution (Figure 2b) [12].

The viscosity of the WPU dispersions decreased with increasing CAB content (Table 1). In general, the viscosity of WPU dispersions shows the opposite trend to the particle size [20]. At the same solid content, the smaller the particle size, the greater the surface area and friction between the particles, leading to a higher viscosity.

### 3.3. Properties of WPU Films

#### 3.3.1. Thermal Properties

**Figure 4:**
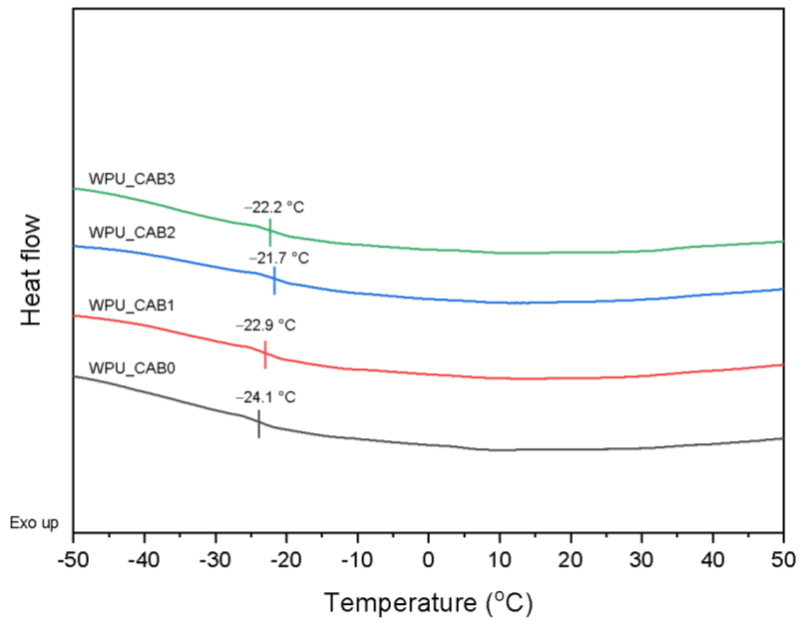
DSC thermograms of WPU films with different CAB contents.

Differential scanning calorimetry (DSC) heating thermograms of WPU films with different CAB contents are shown in Figure 4. All WPU films exhibited a single glass transition temperature, related to the mobility of the soft segment in the WPU component. As the CAB content increased from 0 to 2 × 10^−4^ mol, the *T*_g_ of the WPU soft segment increased from −24.1 to −21.7 °C. This is presumably because the crosslinking points induced by CAB restricted the rotation and movement of polyurethane chains. However, at higher CAB content (WPU_CAB3), the *T*_g_ of the soft segment decreased again to −22.2 °C. This may be because an excessive amount of CAB greatly reduced the hydrogen bonding between the urethane groups, although it increased the crosslink density.

**Figure 5:**
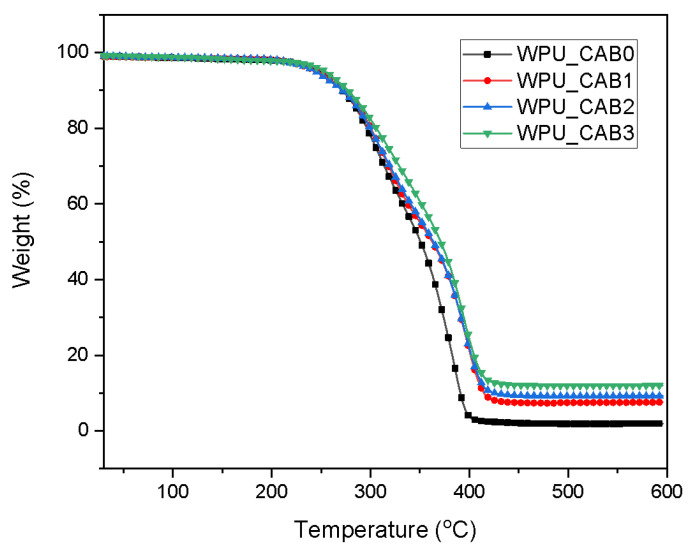
TGA curves of WPU films with different CAB contents.

Figure 5 shows the thermal decomposition behavior of the WPU films as a function of the CAB content. The TGA measurements allow distinguishing the decomposition steps of the hard and soft segments, providing information on the thermal stability associated with the structure of the WPU film. All WPU films underwent three thermal decomposition steps. The weight losses in the ranges of 210–275 and 275–330 °C are due to decomposition of urethane and urea bonds in the hard segments. In general, the hard segments of polyurethane are known to be more susceptible to thermal decomposition than the soft segments. Finally, degradation of the soft segments occurred at 330–400 °C [21]. The retention amounts of WPU films with different CAB contents are shown in Table 3.

As the CAB content increased, the weight loss rate of the WPU films decreased, and their residual amounts at 500 °C increased. This is because a higher crosslinking density of the WPU film results in more energy required to break the structure.

#### 3.3.2. Mechanical Properties

**Figure 6:**
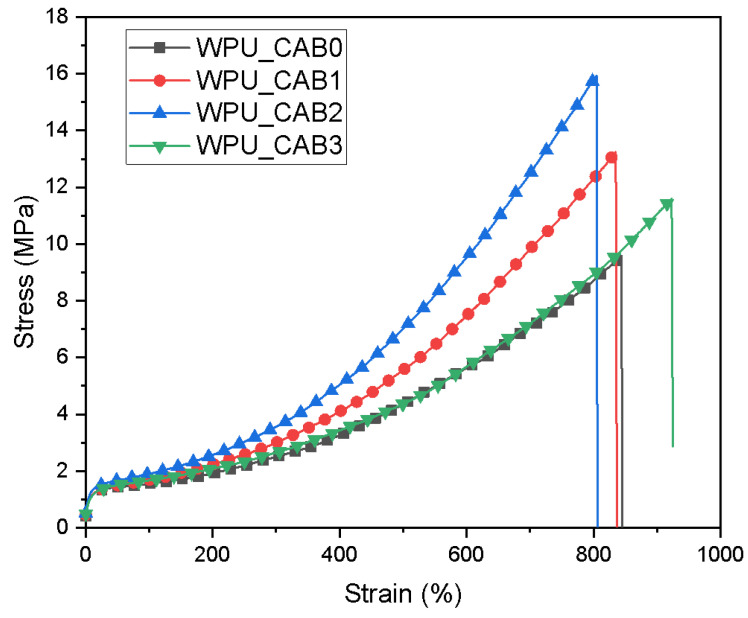
Stress–strain curves of WPU films with different CAB contents.

The stress–strain curves of WPU films are shown in Figure 6, whereas the 300% modulus, tensile strength, and elongation at break of the films are summarized in Table 4. In general, as the crosslink density of an elastic material increases, the tensile strength increases and the elongation at break decreases [22]. As the CAB content increased from 0 to 2 × 10^−4^ mol, the tensile strength of the WPU films increased significantly (from 9.5 to 15.9 MPa), whereas the elongation at break showed a slight decrease, from 845 to 806%. This result is very meaningful, as it shows that the tensile strength improved while the elongation at break remained almost unchanged. These results tended to be different from the tensile properties of polyurethane that were generally chain-extended with low molecular weight chain extenders. The polyfunctional CAB can slightly crosslink the WPU particles, resulting in improved tensile strength of the WPU film. On the other hand, the incorporation of CAB, which is a high molecular weight, can increase the elongation of the WPU film by reducing the strong hydrogen bonding between the polyurethane chains. In the case of WPU_CAB3, the tensile strength decreased and the elongation at break increased again. The appearance of this critical point is due to the reduced interaction between the WPU hard segments owing to the excessive CAB content. The microphase separation of WPU_CAB3 was reduced by the oligomeric nature of CAB, which is unfavorable for bonding hard segments. In addition, the branched polyurethane chains extended using CAB led to a higher degree of intermolecular entanglement, thereby increasing the elongation at break of WPU_CAB3.

#### 3.3.3. Surface Morphologies and Water Contact Angles

**Figure 7:**
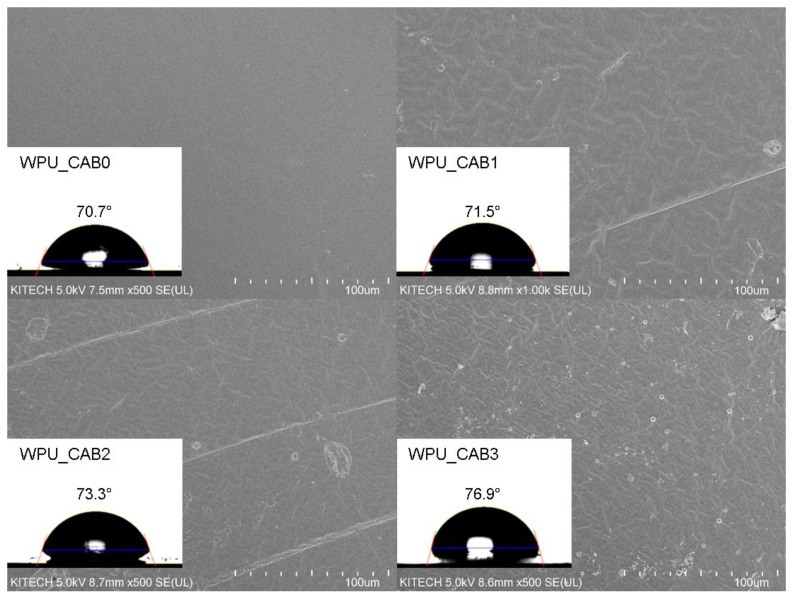
SEM images and water contact angles of WPU films with different CAB contents.

Figure 7 shows the SEM images and water contact angles of the WPU films as a function of the CAB content. Increasing the CAB content resulted in a rougher and more uneven surface morphology of the WPU films. The increased degree of crosslinking caused by CAB induces non-uniform stress during film formation, resulting in a rough surface of the WPU films. Moreover, the particle size of the WPU dispersions can affect the surface roughness of the films [23]. The SEM image of WPU_CAB3 reveals intact particles with a size of 1–3 µm, corresponding to highly crosslinked polymer microspheres that further increased the roughness of WPU_CAB3.

In addition, the incorporation of CAB clearly increased the water contact angle of the WPU films. CAB contains acetyl and butyryl groups that increase the hydrophobicity of the polyurethane chains. As a result, the contact angle of the WPU films increased from 70.7° to 76.9°.

#### 3.3.4. Swelling Behavior

**Figure 8:**
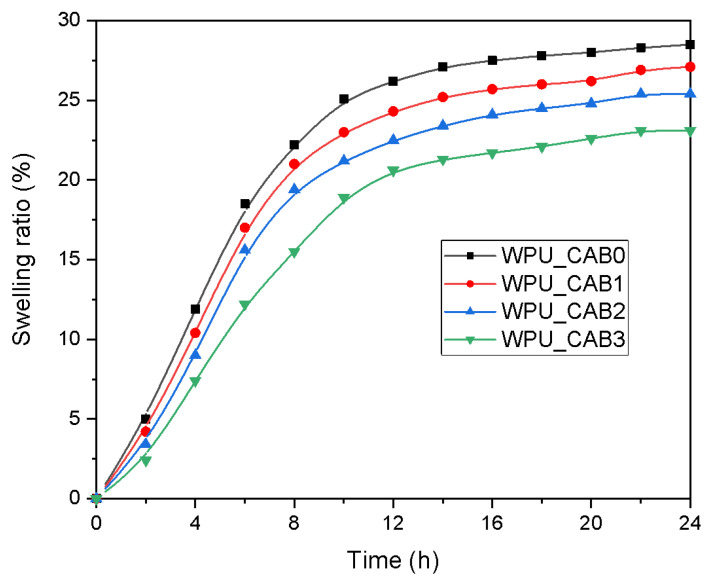
Changes in the swelling ratio of WPU films as a function of time.

The time evolution of the water swelling ratio for WPU films with different CAB contents is shown in Figure 8. The water absorption of all WPU films increased rapidly during the initial 15 h and reached a plateau corresponding to the absorption equilibrium at approximately 15–20 h. The water swelling ratio of the WPU film was significantly reduced upon incorporating CAB into the matrix, with WPU_CAB3 (which had the highest degree of crosslinking) showing the lowest ratio.

#### 3.3.5. Optical Properties

The optical transmittance of WPU films with different CAB contents was evaluated using UV-vis measurements. As shown in Table 4, increasing the CAB content led to a gradual increase in transmittance in the visible region. The polar ester groups of polyesters have a relatively high refractive index and tend to form crystalline domains, which reduce the transmittance of the WPU films. In addition, hard segments such as urethane and urea bonds have a high refractive index and reduce the transmittance of the film [24]. On the other hand, the increase in crosslinking induced by CAB can inhibit the crystallization activity of the soft and hard segments and reduce the formation of hydrogen bonds between the hard segments, thereby increasing the transmittance of the WPU films.

The gloss of the WPU films decreased with increasing CAB content (Table 5). This can be explained by the SEM images in Figure 6. The WPU films incorporating CAB exhibited a rough surface, where incident light at a certain angle was diffusely reflected, reducing the gloss. As a result, the CAB-integrated WPU films were found to have improved matting properties.

## 4. Conclusions

This work highlights the potential of CAB, a renewable resource, to replace conventional petroleum-based chain extenders in WPU systems. An NCO-terminated prepolymer was synthesized using polyester diol, IPDI, and DMPA. The prepolymers were chain-extended using different CAB contents. The proposed structures of CAB and WPU films were confirmed through FTIR spectroscopy. All WPU dispersions had good dispersibility, and CAB increased their particle size and decreased the viscosity. The increase in the degree of crosslinking induced by CAB improved the thermal stability of the WPU films. In addition, the hydrophobicity of CAB increased the water contact angle and decreased the swelling ratio of the films. The CAB-induced crosslinking of the WPU structure produced a WPU film with a rough surface, creating a matting effect. The incorporation of CAB prevented microphase separation of soft and hard segments, resulting in improved optical transmittance. In the tensile properties, the critical content of CAB was observed, because the increase in the chemical crosslink density according to the CAB integration and the decrease in the interaction between the hard segments conflicted. These results prove that CAB contributes to improving the miscibility of soft and hard segments along with the role of chain extension.

## Figures and Tables

**Table 1:** WPU dispersions with different contents of chain extender.

Sample	PBAT (mol)	IDPI (mol)	DMPA (mol)	CAB (mol)	EDA (mol)
WPU_CAB0	0.05	0.1	0.025	-	0.025
WPU_CAB1	0.05	0.1	0.025	1 × 10^−3^	0.021
WPU_CAB2	0.05	0.1	0.025	2 × 10^−3^	0.016
WPU_CAB3	0.05	0.1	0.025	3 × 10^−3^	0.012

**Table 2:** Characteristics of WPU dispersions with different CAB contents.

Sample	Storage Stability (Month)	Average Particle Size (nm)	DPI	Viscosity (cP)
WPU_CAB0	>6	122	0.57	286
WPU_CAB1	>6	147	0.74	124
WPU_CAB2	>6	162	0.76	72
WPU_CAB3	>6	214	0.84	58

**Table 3:** Residual amounts (wt.%) of WPU films.

Sample	Temperature (°C)			
	150	250	350	500
WPU_CAB0	98.3	94.8	50.4	1.9
WPU_CAB1	98.5	94.4	55.1	7.4
WPU_CAB2	98.5	94.1	56.0	9.2
WPU_CAB3	98.3	95.6	60.7	11.9

**Table 4:** Mechanical properties of WPU films.

Sample	300% Modulus (MPa)	Tensile Strength (MPa)	Elongation at Break (%)
WPU_CAB0	2.5 ± 0.1	9.5 ± 0.2	846 ± 22
WPU_CAB1	3.0 ± 0.1	13.2 ± 0.4	837 ± 28
WPU_CAB2	3.6 ± 0.2	15.9 ± 0.6	806 ± 31
WPU_CAB3	2.7 ± 0.2	11.6 ± 0.5	924 ± 30

**Table 5:** Optical transmittance and gloss (60°) of WPU films with different CAB content.

Sample	WPU_CAB0	WPU_CAB1	WPU_CAB2	WPU_CAB3
Transmittance (%)	88.7	91.2	93.1	93.4
Gloss (^o^)	86	76	73	68

## Data Availability

Not applicable.
